# A marketing perspective on consumer perceived competition in private ophthalmology services

**Published:** 2018

**Authors:** Consuela-Mădălina Gheorghe, Victor Lorin Purcărea, Iuliana-Raluca Gheorghe

**Affiliations:** * “Carol Davila” University of Medicine and Pharmacy, Bucharest, Romania

**Keywords:** consumer perceived competition, private ophthalmology services, price in health care services, satisfaction in health care services

## Abstract

Competition in health care services has been considered as a core component in the consumer value mechanism. In Romania, the emergence of private ophthalmology services has as outcome a positive-sum competition which focuses on prevention, diagnosis and treatment of eye related diseases and disorders and improved value.

The purpose of this paper was to provide an insight into the knowledge used by consumers when they make a decision regarding a private ophthalmology service with a specific interest on competition.

A research model was elaborated and included quality, price, and satisfaction as components influenced by competition from the consumers’ perspectives. The model was validated using Structural Equation Modeling in SmartPLS. The sample was made up of 120 respondents and the sampling technique was quota.

The structural model revealed that competition has a positive impact on satisfaction and explained 74% of its variance and also that competition has a positive impact on price and explained 7% of the variance. Moreover, using IPMA matrix analysis, the most powerful item of competition construct with an influence on satisfaction was the one related to the reputation of the private ophthalmology organization.

Nonetheless, the key to gain competitive advantage stands in providing meaningful value to consumers using differentiation features such as high prices, reputation, and awareness on the services offered, greater availability, and the most important feature, innovation.

## Introduction

Competition in health care has an important role in the delivery and financing of every health care system. For example, empirical studies in the United States have had a very strong focus on the hospital competition within their countries, suggesting there is a strong connection between competition and quality in health care services [**1**, **2**]. More exactly, these studies measure hospital competition using the sum of squared market share for all hospitals included in a particular geographic area, calculating the number of hospital discharges divided by the total discharge rate in a given geographic area [**1**]. However, measuring hospital competition is a subject of disagreement in many empirical studies [**3**].

Considering the fundamental role of competition in Marketing Management, experts analyzed competition related issues from an external organizational perspective, as well as the internal organizational perspective, in order to understand the outcomes of organizational competitive behavior [**4**]. Many theoretical frameworks have grounds in the internal capabilities of the organization such as the resource-based view [**5**] and dynamic capabilities [**6**], but the dominant competition theories have been implemented with a particular external approach, being inspired by industrial economies [**7**], game theory [**8**] and the network theory [**9**]. While the IO-Economics perspective provided by Porter [**7**] focuses on the identification of external factors such as characteristics and macro-economic conditions that would determine positive performance outcomes, current research shifted towards the competitive dynamics environment [**10**]. Hence, competitive dynamics examines the interaction of the internal and external environment with competition; the core elements of competitive dynamics being the identification and analysis of implied competitive actions. 

Traditional competition in health care services involves more than one element such as price, quality, convenience superior services, new technology and innovation. However, the key role of competition in health care is its potential to provide a mechanism for reducing health care costs. More exactly, competition offers the possibility to eliminate inefficiencies that would lead to high production and delivery costs, which are transferred to patients in high health service prices in the private sector and low insurance coverage in the public sector. Moreover, Baker explained that the following important issues should be included in measuring health care competition: the services offered, market areas, basic measurement forces that modify the competitive dynamics [**11**]. As such, competition should be carefully measured by identifying the services as well as the organizations and the relevant geographic market area. Obviously, the proportional increase in competition brings an increase in the number of organizations as well.

It must be acknowledged that the health care market is imperfectly competitive, suggesting that most markets are oligopolistic and monopolistic, caused by several factors such as entry barriers, asymmetry of information, monopoly power, non-uniformity in health care quality, common motivations other than profit and a high degree of uncertainty [**12**]. Due to the imperfections in competition, Miller and Porter and Teisberg noted a price based orientation among hospitals and physicians [**13**, **14**]. Further, the network style of care and even the delivery of increased consumer value are taken into consideration in health care services. However, the fundamental pillar of constant quality improvement and cost decrease is innovation [**15**]. It is still under great debate whether the cost savings from price competition are a result of improved efficiency against quality services [**16**]. Undoubtedly, competition in health care should be initiated in the shape of technology differentiation with the anticipated implementation and service outcome of patient satisfaction. 

According to the Romanian Institute of Statistics, in 2015 there were 1723 public ophthalmology organizations with 130 one-day hospital beds and 35 private ophthalmology organizations with 36 one-day hospital beds. In contrast, in 2016 there were 1695 public ophthalmology organizations with 130 one-day hospital beds and 38 private ophthalmology organizations with 54 one-day hospital beds. In addition, around 2015 there were 776 specialized physicians in Ophthalmology in public organizations and 467 specialized physicians in private organizations. Moreover, in 2016, the number of specialized physicians in Ophthalmology increased with 22.12% in the public health care organizations and with 23.56% in private health care organizations, as illustrated in **Table 1**. Further, according to the Romanian Society of Ophthalmology, there were 965 active members in 2017, which led to the conclusion that the Ophthalmology specialty is a field of great interest and will continue to grow [**17**]. Thus, this research aims to provide an insight into the knowledge and practice related to ophthalmology competition from a marketing perspective. More exactly, the research determined the knowledge used by health care consumers when they make a decision regarding ophthalmology services with a specific interest in the role played by competition [**18**].

**Table 1:** The distributions of Romanian physicians and health care organizations specialized in Ophthalmology

Ophthalmology Organizations Ophthalmology Physicians	Year		
	Type of Organization/ Physician	2015	2016
	Public	1723	1695
	Private	35	38
	Working in Public Organizations	776	1217
	Working in Private Organizations	467	755

This paper is structured as follows: the background section presents the relevant literature on price satisfaction, quality of care and satisfaction in health care services as well as the impact of ophthalmology services competition on the aforementioned variables from a consumer’s perspective. Consequently, a research model of consumer perceived competition in a private ophthalmology organization is described in Material and Methods section, followed by the findings and the discussion sections. 

## Background

Literature review on competition in Ophthalmology services

As one of the foundational pillars of Strategic Marketing, competition has been the focus of various inquiries and analysis [**19**,**20**]. Determining a suitable place in an intensive and dynamic competitive environment is the key to ensure long-term profitability and survival in any business. For instance, assessing this objective is attainable only through creating and keeping a competitive advantage [**7**]. According to Porter, competitive advantage refers to a “set of capabilities that permanently enable the business to demonstrate better performance than its competitors” [**7**]. Moreover, competitive advantage may be achieved in three ways: cost leadership, centralization and creating a differentiated service [**21**].

**Fig. 1:**
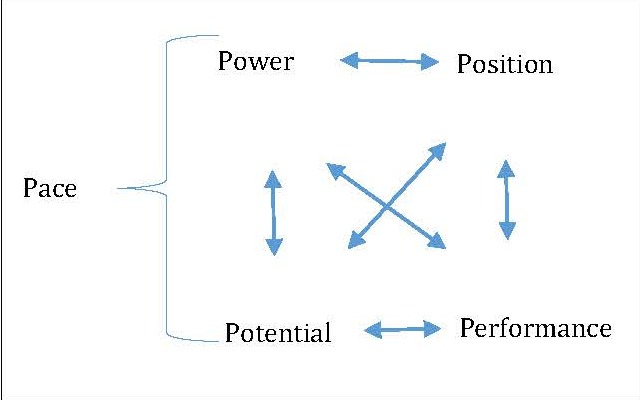
Five major sources of competitive advantage [**22**], p. 22

There are as many sources of advantage as there are markets or competitors. Those sources can be grouped into categories for studying them. Luke, Begun and Walston labeled with “P” all categories of competitive advantage sources [**22**]. As such, the first phase of the early entrant is pace and the basis for an advantage is the measurement of timing and intensity of the strategic action, while lowering costs. The outcomes of pace are expanding an organization’s market shares, increase economies of scale and market power. The second competitive advantage source is Position, which encompasses a projection of distinctive and valued image to consumers whereas Potential materializes in the shape of access to distinctive and superior capabilities and resources. Consequently, Performance consists of superiority in operations, as well as the implementation of strategy, and, last but not the least, Power, which represents the accumulation and the efficient usage of organizational mass. As such, the second phase consists of efficiency gains if the organization reached its maturity stage. Thus, this phase enables a competitor to define its position on the market by lowering its costs, using at the same time, the positioning competitive advantage. Nevertheless, alternative sources of competitive advantage may focus on building up consumer loyalties or switching costs, both strategies being related to positioning and power. **Fig. 1** illustrates the sources of competitive advantage and the interaction between them. 

In health care services, competition remains a vital component of any marketing plan as it relates to economic growth. Porter and Teisberg advocate that there are three elements of competition, namely, physicians and other practitioners, organizations that provide health care services in any shape and organizations that provide health care financing and insurance plans [**14**]. 

Greenberg concluded that physicians might compete for patients both with and without insurance, for non-price basis elements such as location, referrals, and reputation, for affiliation with a preferred provider organization [**23**]. Further, health care organizations, meaning, hospitals, hospital systems, and other health services organizations may compete for physicians, third party payers and, of course, patients [**24**]. 

Today, most health care organizations have the tendency to compete for patients by offering and providing more services, which are not basic ones, amenities, and even discounted prices [**25**]. Competition among health insurance plans and their coverage expenses is very complicated and difficult to be determined by patients. Similarly to the reported situation in the United States by Porter and Teisberg, in Romania the competition in public health care organizations has become zero-based, suggesting that participants involved in health care delivery services are engaged in dividing value rather than creating it through investing in unnecessary costs. The particular features of zero-based competition is associated with an incorrect level of competition and geographic market and focus on cost reduction and satisfaction surveys as well as offering improper financial rewards to providers [**14**]. However, in private health care organizations, also referring to private ophthalmology organizations, Porter and Teisberg identified a positive-sum competition characterized by prevention, diagnosis and treatment of specific diseases, eye diseases, respectively, improved value, adopting a suitable level of competition and a suitable market offer and complete information about providers, treatments and alternatives in the case of specific conditions [**14**]. Moreover, we believe that the positive-zero based competition may be successfully achieved if its features are measured and validated by consumers. As such, while the phenomenon of competition has been heavily studied, various opportunities aroused in extending and contributing to the existing literature by examining competition and its established relationships with other consumer specific factors as quality and satisfaction. Obviously, competition in health care services compels providers to deliver increased value and this continuous quality improvement and cost reduction is supported by innovation [**15**]. 

Literature review on perceived quality in Ophthalmology services

The key to achieve sustainable and intense competitive advantage on today’s markets lies in delivering high quality services that will have as an outcome satisfied consumers. 

Specialists in Marketing and Management have tried to find a concise and meaningful definition of quality, especially in the health care field, in which the concept is applied differently, from medical specialty to medical specialty. Generally, from a consumer perspective, Fornell et al. defined service quality as the discrepancy between the expected service and the perceived service [**26**]. When the expected service is higher than the perceived service, the perceived service quality is less than satisfactory, whereas when expected service equals the perceived service, the perceived quality is satisfactory, and, when the expected service is lower than the perceived service, the perceived quality is more than satisfactory and will tend toward an ideal quality [**27**]. 

In health care services, quality is “the production of improved health and satisfaction of a population within the constraints of existing technology, resources, and consumer circumstances [**28**]. Moreover, health care services are credential in nature; therefore, consumers are unable to determine the technical quality of the service. The entropy of information shapes the functional quality of the health care service, being represented by the quality of interaction between physicians and patients, the quality of communication, physician’s ability to maintain the patient’s trust and his skills to treat patients with sensitivity, empathy and concern [**29**].

Further, quality in private ophthalmology services was measured with the SERVQUAL scale [**30**]. The dimensions that define the SERVQUAL scale are [**27**]:

- Tangibles encompasses the physical aspect of the organization, the equipment used and the appearance of both medical and auxiliary personnel;

- Reliability is shaped by the providers’ ability and skills to deliver the service as accurately as promised;

- Responsiveness focuses on the personnel’s willingness to provide a prompt service;

- Assurance is made up of the personnel’s knowledge, courtesy and skills to inspire trust and confidence;

- Empathy consists of the personnel’s skills to provide caring and personalized delivery of services. 

Using the gap score analysis, suggesting the difference between the expected service and the perceived service, Gheorghe et al. reached the conclusion that the highest gap score in ophthalmology services was registered by the Tangibles dimension, whereas the lowest gap score was registered by Reliability dimension [**30**]. 

*Literature review on satisfaction in Ophthalmology services*

For some experts, satisfaction should be the ultimate outcome when applying marketing strategies. For the highly competitive health care market, satisfaction is not only a monitor of improvement but also a feature that may attract patients. 

It is acknowledged that there is no consensus in the conceptualization and the relationship between satisfaction and quality, even in health care services. In the services literature, satisfaction is considered both a psychological and encounter state [**31**], an outcome state [**32**], an experiential construct [**33**] and is depicted as a discrepancy between prior expectations and actual performance [**34**], as well as having both an affective and a cognitive concept [**32**]. Moreover, there are varieties on the subject when measuring satisfaction, in fact, it depends on the research objectives [**35**]. Thus, the satisfaction construct may be employed in two ways, namely, as a dependent variable that serves as an indicator of the structure, process, and outcomes activities, or it may be an independent variable implemented in predicting consumer behaviors [**35**]. 

In the health care environment, Ware et al. stated that satisfaction should be a multidimensional concept that includes “the art of care” (the personality features of the physician), technical competence (consumer’s perception of the physician’s knowledge and expertise), the physical environment in the consumer’s perspective, as well as the efficacy of care (the consumer’s perception of the outcome) [**36**]. As it can be observed, many elements are also found in the quality construct. In fact, there is no consensus regarding the way to adequately describe the relationship between quality and satisfaction, with a specific emphasis on health care services. Some experts (i.e. [**37**]) concluded that it is more relevant to measure the functional and technical perceived qualities rather than determining patient satisfaction. Gotlieb et al. uncovered a clear distinction between perceived service quality and satisfaction in health care services [**38**]. In addition, Taylor highlighted that in health care services a confusion between the two constructs occurred and they were employed interchangeably [**39**]. In this paper, we considered consumer satisfaction as an independent construct but as an outcome of service quality [**40**]. 

*Literature review on price in Ophthalmology services*

The established relationship between competition and health care system costs has indicated contradictory results, meaning that there is not enough evidence to prove that higher prices would decrease or increase the satisfaction levels from consumer perspectives as well as whether a cost decrease would have as beginning point of a low strategy pricing.

In private health care services, it is known the fact that most organizations implement a price based competition but this strategy would make them more cost-effective and deliver lower quality services despite the enjoyment of consumers. 

There are five dimensions of price that are worth mentioning [**41**]:

- Price transparency should provide clear, comprehensive, current and effortless information about the price overview of an organization;

- Price quality ratio reflects a trade-off between the perceived quality of a service and the monetary costs involved;

- Relative price reflects the price offer compared to that of the competition;

- Price confidence focuses on the consumer’s certainty that the price is favorable;

- Price reliability consists of the perceived prevention of negative price surprises;

- Price fairness reflects the consumer’s perception of whether the difference between the price accepted by society in comparison to another party is acceptable and justifiable. 

**Fig. 2:**
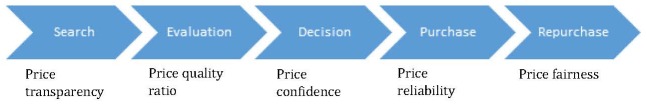
Price dimensions classified according to the consumer’s decision-making process

Further, Mittal et al. pinpointed that the correct price dimension analysis depends on the decision-making stages of the consumer [**42**] (**Fig. 2**).

In health care services, we believe that most consumers decide on price criteria depending on their perceived risk. If the consumer has a high level of perceived risk, he/ she will make a price confidence decision, whereas if the level of risk is low, the consumer will make a price quality ratio decision. No matter the price decision made, every private ophthalmology organization in Romania applies a price transparency strategy. 

The conceptual framework of the research model

**Fig. 3:**
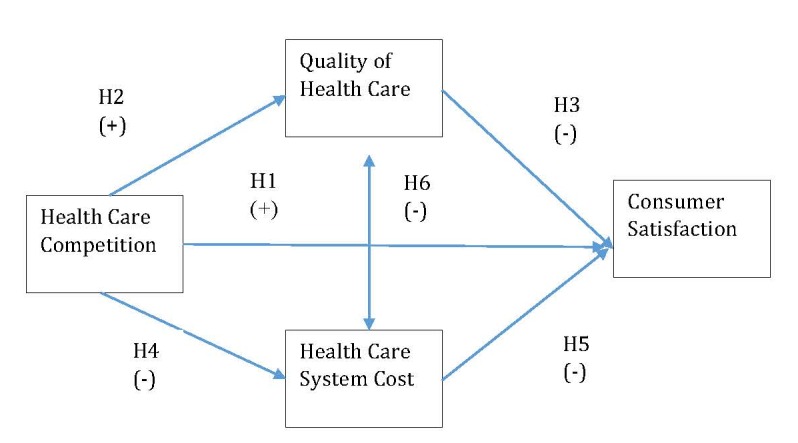
Competition and consumer satisfaction research model [**43**]

Asoh and Rivers elaborated a research model of health care competition and consumer satisfaction [**43**]. In their model, they included the following factors that are influenced by competition: quality of health care, health system cost and consumer satisfaction (**Fig. 3**). In line with the conceptual framework, they conceived the following hypothesis [**43**]:

1. As the level of competition within the health care market increases, the level of consumer satisfaction increases. 

2. As the level of competition within the health care market increases, the quality of health care provided to consumers increases. 

3. As the quality of health care provided to consumers increases, the level of consumer satisfaction increases. 

4. As the level of competition within the health care market increases, the level of health care system costs decreases. 

5. As the level of health care system costs decreases, the level of consumer satisfaction increases. 

6. As the level of health care system costs increases, the quality of the health care provided to consumers increases. 

In our research model we kept the variables competition, quality and satisfaction, but we renamed health care system costs with price satisfaction, since we are focusing on a private ophthalmology organization, in which individuals come and pay for a consultation directly without involving a third party insurance organization. Based on Asoh and Rivers’ model [**43**], we elaborated the following hypotheses:

1. As the competition level in ophthalmology services increases, the level of perceived quality increases as well;

2. As the competition level in ophthalmology services increases, the perceived satisfaction increases as well;

3. As the competition level in ophthalmology services increases, the perceived price satisfaction increases as well;

4. As the perceived service quality in ophthalmology services increases, the perceived price satisfaction increases as well;

5. As the perceived price satisfaction increases in ophthalmology services, the perceived satisfaction increases as well;

6. As the perceived quality in ophthalmology services increases, the perceived satisfaction increases as well. 

**Fig. 4:**
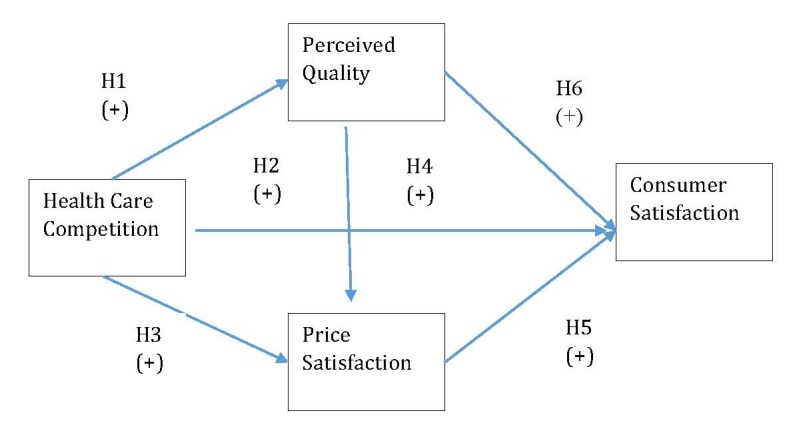
Conceptual framework of the research model in private ophthalmology services

The conceptual framework of our research model is depicted in **Fig. 4**.

## Material and methods 

In line with Asoh and Rivers’ model [**43**], we elaborated a conceptual framework that would offer the possibility to conduct an in-depth investigation of competition in private ophthalmology services from a consumer perspective. Consequently, we included in our model, factors such as competition, price satisfaction, perceived quality, and satisfaction. Despite the fact that Asoh and Rivers described their model with great details, they did not include any clues on the empirical side of any research. In fact, as further research, they recommended the model to be empirically tested. 

The data collection phase consisted of 2 stages: a pilot study and the validation of the model. In the pilot study stage, we developed a self-administered questionnaire with some items found in the scientific literature in order to eliminate any biased questions. The competition construct was made up of several items referring to technology, level of service quality delivery, professional standard services, infrastructure, variety of services, reputation of the organization, price and ease of obtaining information, whereas the perceived quality construct included items of the SERVQUAL measuring scale [**30**]. The price satisfaction construct followed the price classification of Mittal et al. [**42**] and items referring to complete, accurate and comprehensive prices plans were included, and last, but not the least, satisfaction encompassed items indicating the perceived emotions post-experience such as delight and trustworthiness. All the items included in the questionnaire were adapted according to the private ophthalmology services context and were measured on a 5-point Likert scale ranging from 1-Totally Disagree to 5-Totally Agree. The pilot sample size for testing the questionnaire was made up of 40 respondents and we reached the conclusion that no amendments were required. 

The validation stage of the model was assessed in SmartPLS version 3, using Partial Least Squares Technique. The sample of respondents was made up of 120 individuals who were selected during a 3-day period. The selection was conducted on a quota-sampling basis, suggesting that every fifth patient of the private ophthalmology organization received a questionnaire. The final version of the questionnaire was structured on 2 sections: the first section collected demographic data of the respondents such as gender, age and the second section comprised the factors’ items included in the model. 

The statistical analysis was performed in SPSS version 20 in order to determine the demographic profile of the respondents and in SmartPLS version 3 the validation of the structural model. The Partial Least Squares (PLS) technique is a multivariate analysis that involves the simultaneous investigation of complex relationships and is commonly used in theory development [**44**]. In addition, PLS-SEM works efficiently on small samples and has greater statistical power [**45**]. Moreover, following the PLS analytical process stages, first a psychometric investigation assessment of the constructs’ scales is required, followed by the validation of the structural model [**44**]. 

## Findings

• Demographic profile of the respondents

The respondents were asked to fill in the questionnaire with their details about gender, age, level of education, monthly income, and type of consultation. Among the 120 respondents, there were 45.8% females and 54.2% males, who graduated a university institution (36.7%) and with a monthly income greater than 30001 RON (40.8%). The reasons for seeing a physician specialized in Ophthalmology were routine check-ups (44.2%) and eye analysis (43.3%). 

• Measurement model

The validity assessment of the research model was determined by the values of three coefficients, as follows: Cronbach’s alpha coefficient, which measures the internal consistency reliability of the answers provided by the respondents, followed by the Convergent Validity Coefficient and the Variance Extracted Value. All these coefficients should have values greater than 0.7 and the AVE should be greater than 0.50 [**44**]. Consequently, the psychometric values of the coefficients as well as their factor loadings are illustrated in **Table 2**. Factor loadings should have values greater than 0.5. It is worth mentioning that perceived quality construct in the beginning of the process analysis, was a second order factor, but, since the latent constructs of Tangibles, Assurance, Empathy and Responsiveness did not load properly on the second order factor, we concluded that the perceived quality construct should be transformed in a first order factor, containing only the Reliability scale items. As it can be observed in **Table 2**, all conditions to achieve a robust measurement model were satisfied. 

**Table 2:** Psychometric values of the latent constructs

Construct	Item	Loading	CV/AVE/Cronbach’s alpha
Perceived Quality (Reliability)	It1	0.82	0.93/0.79/0.91
	It2	0.86	
	It3	0.86	
	It4	0.89	
	It5	0.88	
Competition	It1	0.79	0.95/0.64/0.94
	It2	0.84	
	It3	0.80	
	It4	0.82	
	It5	0.78	
	It6	0.80	
	It7	0.80	
	It8	0.80	
	It9	0.80	
	It10	0.79	
	It11	0.80	
Price satisfaction	It1	0.87	0.93/0.74/0.91
	It2	0.85	
	It3	0.86	
	It4	0.85	
	It5	0.85	
Satisfaction	It1	0.89	0.90/0.75/0.83
	It2	0.85	
	It3	0.85	

• Structural model

**Fig. 5:**
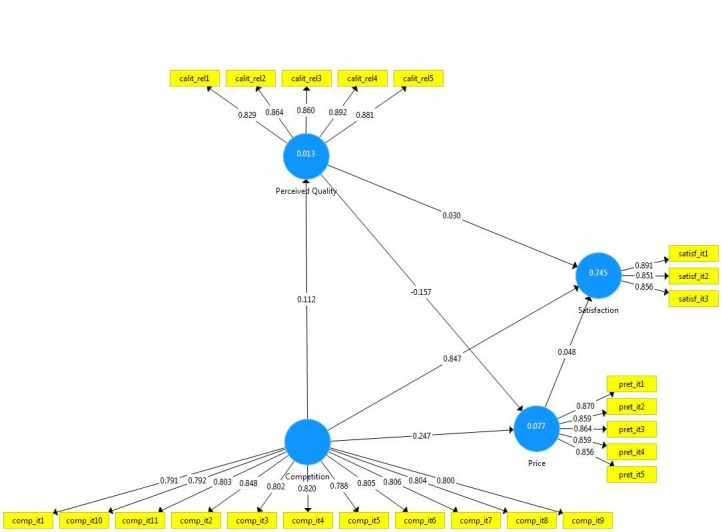
The Structural Model and the path coefficients

The Bootstrapping procedure was employed to determine the structural model. As such, the significance of the estimated path coefficients at a p value lower than 0.05 as well as the value of explained variance (R2) revealed that H3 and H2 are supported, suggesting at the same time that competition explained very much the variance of satisfaction (74%) and competition explained substantially the variance in price (7%) (**Fig. 5**).

• Importance Performance Map (IPMA)

The Importance performance matrix analysis is extremely useful in extending the outcomes of the PLS-SEM model based on the latent variable scores [**44**]. Determining the IPMA accurately brings advantages at a managerial level, as many strategies should focus on a latent construct’s items. 

**Fig. 6:**
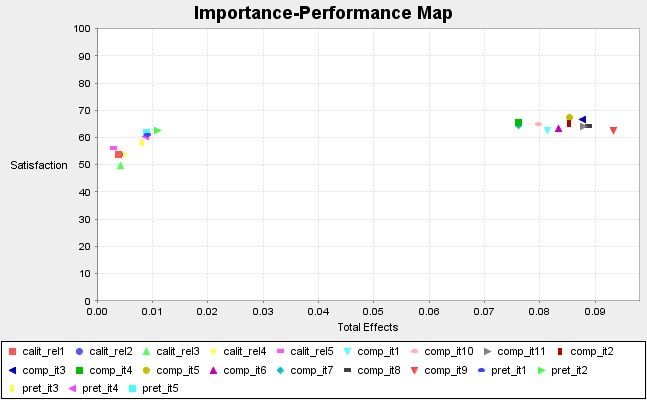
The IPMA of competition and satisfaction

In our model, the most powerful item of the competition construct with an influence on satisfaction was the one related to reputation (**Fig. 6**).

## Discussion

Specialists in health care competition pinpointed that this market is based on price as the fundamental pillar but from the perspective of consumers, competition brings quality, reputation and experience choices. 

For many years, Romania was under socialist governance, leading to a monopolist competitive market characterized by public health care organizations. Consequently, after the fall of the socialist regime, the first private organizations related to health care were offered by dentists and general practitioners, while other medical specialties started to be introduced after the year 2000 [**46**]. Although, the Romanian private sector has an increasing trend and it has not reached its maturity stage, most services are delivered at the level of the 1980s’ developed countries, the main sources of speeding up the process being the integration of technology and IT as well as the number of qualified personnel available [**46**]. 

According to Porter’s competitive strategies, there are three approaches of performing in any industry: overall cost leadership, differentiation, and focus [**47**]. Each strategy is integrated within a modern marketing theory framework, keeping in mind that each generic strategy is fundamentally a different approach to creating and sustaining a competitive advantage, as illustrated in **Table 3** [**48**]. 

**Table 3:** Three Generic Strategies [**47**], p. 4

Competitive Scope		Competitive Advantage	
		Lower Cost	Differentiation
	Broad Target	1. Cost Leadership	2. Differentiation
	Narrow Target	3A Cost focus	3B Differentiation Focus

In our research, we identified the powerful relationships established between competition and satisfaction as well as competition and price satisfaction in private ophthalmology organizations as perceived by consumers. Moreover, the established relationships revealed that if the competition is perceived as high, the satisfaction would also register high levels and if competition level were perceived as high, the price satisfaction would register high levels. As such, competition in health care services, as well as in ophthalmology services, can take place at several bases such as price, consumer choice of providers, access, style of care and technical quality of care [**13**]. In this context, it is worth mentioning that “competition on the basis of price” refers only to the service’s price attribute and no other attributes relating to quality or satisfaction. It is true that consumers are price sensitive but a vast number of academic studies suggest that selecting a choice is grounded on the decisions of consumers. 

## Conclusions

Differentiation can be determined by a vast palette of features such as better quality, lower price (high price), consumer service, reputation, provide greater awareness on the services offered and greater availability. The key stands in the adequate identification of different features that will provide a meaningful value to consumers to gain competitive advantage [**47**]. In health care services, the competitive advantage factors evolved over time, being shaped by the forces present in a market at a certain point in time. As such, the differentiators from the past, as for example, the infrastructure, the service attributes and the human resources become qualification factors in the present, as for instance competitive capabilities, the elements of strategy and IT technology. However, what makes the difference in the shape of competitive advantage refers to competitive capabilities, and the top three choices of Romanian consumers are doctors, medical technology, and the waiting time for accessing the health care service [**46**]. Further, other mentioned competitive capabilities for health care services are the range of medical specialties in the same location, the hours when the organization is open, the ease of scheduling appointments, the ease of reaching the medical staff, the thoroughness and accuracy of the diagnosis, developing systems that ensure quick and easy appointment scheduling, communication channels with patients, comfortable facility with additional benefits (free parking places or internet access), basically consumer centric services. 

In the case of low cost strategy, high prices should be complemented by fast access to medical appointments, preventive medicine, regular check-ups, and standard services in order to increase the number of patients [**46**]. Further, the most influential item of competition on satisfaction relates to the reputation of the organization. The strategy is called focused differentiation and targets patients with highly specialized diseases, so consumers will come mainly from other people’s referrals. 
